# Algorithm for the treatment of external nasal valve insufficiency^☆^

**DOI:** 10.1016/j.bjorl.2019.02.008

**Published:** 2019-04-23

**Authors:** Eduardo Landini Lutaif Dolci, José Eduardo Lutaif Dolci

**Affiliations:** a Santa Casa de São Paulo, Faculdade de Ciências Médicas, São Paulo, SP, Brazil; b Santa Casa de Misericórdia de São Paulo, Departamento de Otorrinolaringologia, São Paulo, SP, Brazil

**Keywords:** Nasal obstruction, Rhinoplasty, Nasal surgery, Obstrução nasal, Rinoplastia, Cirurgia nasal

## Abstract

**Introduction:**

Nasal obstruction is one of the most prevalent complaints in the population. The main causes of nasal obstruction are inflammatory, infectious or anatomical alterations. Anatomical alterations include nasal septum deviation, turbinate hypertrophy, and nasal valve insufficiency (external and/or internal). The diagnosis of nasal valve insufficiency remains a clinical one and is based on inspection and palpation of the nose, evaluating both its static and dynamic functions. The literature presents several options for the correction of external nasal valve insufficiency. These are chosen according to the choice and experience of each surgeon.

**Objective:**

To create a practical algorithm for the treatment of external nasal valve insufficiency that can guide nasal surgeons in their choice of treatment for the different anatomical alterations found in patients with these disorders.

**Methods:**

We used the treatment options found in the literature and correlated them with our surgical options for each type of anatomical alteration found. Therefore, we used basically three parameters related to physical examination findings (degree of insufficiency and characteristics of the lower lateral cartilage) and the patient's complaint (present or absent aesthetic complaint regarding the nasal tip).

**Result:**

A practical algorithm was developed for the treatment of external nasal valve insufficiency according to the degree of insufficiency (mild-to-moderate or severe), aesthetic complaint of the nasal tip (present or absent) and characteristics of the lower lateral cartilage (size and orientation).

**Conclusion:**

Through this simple algorithm, one can use each type of graft and/or maneuver according to the patients’ complaints and the anatomical alterations found.

## Resumo

**Introdução:**

A obstrução nasal é uma das queixas mais prevalentes na população. As principais causas de obstrução nasal são inflamatórias, infecciosas ou alterações anatômicas. As alterações anatômicas incluem: desvio do septo nasal, hipertrofia de conchas nasais e insuficiência da válvula nasal (externa e/ou interna). O diagnóstico da insuficiência de válvula nasal permanece clínico e baseado na inspeção e palpação do nariz, avaliado em funções estática e dinâmica. Temos na literatura diversas opções de correção da insuficiência de válvula nasal externa. Essas são escolhidas de acordo com a opção e experiência de cada cirurgião.

**Objetivo:**

Criar um prático algoritmo para o tratamento da insuficiência de válvula nasal externa, que oriente os cirurgiões de nariz na escolha do tratamento para as diferentes alterações anatômicas encontradas nos pacientes portadores dessas alterações.

**Método:**

Utilizamos as opções de tratamento encontradas na literatura e relacionamos com as nossas opções cirúrgicas para cada tipo de alteração anatômica encontrada. Dessa forma, utilizamos basicamente três parâmetros relacionados aos achados de exame físico (grau de insuficiência e características da cartilagem lateral inferior) e à queixa do paciente (queixa estética da ponta nasal presente ou ausente).

**Resultado:**

Um algoritmo prático para o tratamento da insuficiência de válvula nasal externa, de acordo com o grau da insuficiência (leve-moderada ou severa), a queixa estética da ponta nasal (presente ou ausente) e as características da cartilagem lateral inferior (tamanho e orientação).

**Conclusão:**

Através desse simples algoritmo, podemos utilizar cada tipo de enxerto e/ou manobra de acordo com as queixas dos pacientes e as alterações anatômicas encontradas.

## Introduction

Nasal obstruction is one of the most prevalent complaints in the population. The main causes of nasal obstruction are inflammatory conditions, anatomical abnormalities and infectious processes. Anatomical alterations include nasal septum deviation, turbinate hypertrophy, and nasal valve insufficiency (external and/or internal). In the last decades, the improved evaluation of the nose and a better understanding of nasal anatomy and physiology heightened attention to this region during nasal surgeries, both for preventing these alterations during purely esthetic surgeries and in surgical procedures performed for treatment. Nasal valve insufficiency has been diagnosed as the cause of nasal obstruction in up to 13% of adults.^1^ Additionally, 95% of patients with persistent nasal obstruction after septoplasty have the nasal valve as a responsible factor.^2^

In general, three structures make up the nasal valve region: inferior turbinate, nasal septum and lateral nasal wall. The first two are static and rigid structures, whereas the latter, less rigid, is a variable determinant for nasal valve stability. Therefore, it is important to diagnose which of these structures are responsible for adversely affecting the nasal valve function.^3^

The nasal valve is comprised of two anatomically close regions, which can be responsible for nasal valve failure, either alone or together. The internal nasal valve is an angle formed medially by the upper portion of the nasal septum, superiorly and laterally by the caudal portion of the upper lateral cartilage and inferiorly by the head of the inferior turbinate.^4^ In Caucasian noses, this angle varies between 10° and 15°. The external nasal valve is constituted medially by the caudal septum and columella, superiorly by the weak triangle, and laterally by the alar rim (caudal rim of the lateral crus of the inferior lateral cartilage) and inferiorly by the nasal vestibule floor.^5^

The main complaint of patients with nasal valve insufficiency is difficulty in obtaining adequate passage of air through the nose. In the literature the diagnosis remains subjective, and there is no gold standard test for this diagnosis to date. The clinical history associated with otorhinolaryngological physical examination, anterior rhinoscopy, and external inspection/palpation of the nose are important for this evaluation. Complementary examinations, such as rhinomanometry and nasofibroscopy, are less useful for evaluation and diagnosis of nasal valve insufficiency.^6^

One study demonstrated that the use of external nasal dilators may be useful in confirming the diagnosis, allowing differentiation of the affected site (lower lateral and/or upper lateral cartilages). For this purpose, this device must be positioned over the nasal wing (lateral crus of the lower lateral cartilage) or over the cartilaginous nasal dorsum (caudal portion of the upper lateral cartilage), and then verifying in which situation an improvement in the obstruction sensation occurs.^7^

The performance of the modified Cottle maneuver has also been effective in the functional rhinoplasty surgical programming, and is more specific than the traditional Cottle maneuver. In the traditional maneuver, the cheek region is drawn laterally with one or two fingers, checking for obstruction improvement. This maneuver does not allow the individual evaluation of the internal or external valves.^8^ In the modified Cottle maneuver, a metal stylus, or even an otological curette, is used to laterally push the upper or lower lateral cartilage region, verifying in which situation there is airflow improvement. Therefore, the maneuver allows the isolated evaluation of each region.

### External nasal valve

External nasal valve insufficiency is related to either congenital alterations of the structures that constitute this region, or alterations that were acquired after a previous nasal surgery (iatrogenic). Congenital alterations related to functional problems are fragile cartilages susceptible to collapse during inspiration or poorly positioned lower lateral cartilages^9^ (in an inadequate cephalic or sagittal position, in which the caudal rim of the lateral crus is at a different level relative to the cephalic rim).

The correct definition of the anatomical alteration site is essential so that appropriate actions can be undertaken – columella, caudal septum, alar rim (congenital or iatrogenic lateral crus fragility), or a combination of these.

There are no doubts about the treatment when caudal septal deviations or a large (obstructive) columella are found. In these situations, septoplasty and columelloplasty are the treatments of choice. However, when alterations in the lower lateral cartilages are found, several options have been described.

The main surgical options for correction are: Batten graft; Alar rim, Articulated alar rim graft; Lateral crural strut graft; Lateral Crural Turn-in Flap; Seagull wing graft; and Lateral crural graft.^10^, ^11^, ^12^, ^13^, ^14^, ^15^, ^16^

## Methods

The choice of the type of graft used in the correction of lateral crus alterations should be defined by the materials available for grafting, the degree of the alteration found and, especially, by the experience and preference of each surgeon. Therefore, we have created a practical algorithm for the treatment of external nasal valve insufficiency. We did not find in the literature any articles that addressed this practical implication. We found only one article that addresses the authors’ treatment protocol.^17^ However, they use only one type of graft, the batten graft, for the correction of the entire valvular region (internal and external valve).

Thus, our aim is to allow surgeons who have recently started performing nasal surgeries, specifically in functional and esthetic rhinoplasties, to have treatment options according to the anatomical alterations found, and according to the availability of grafts for each patient as well.

## Results/discussion

**Figure 1:**
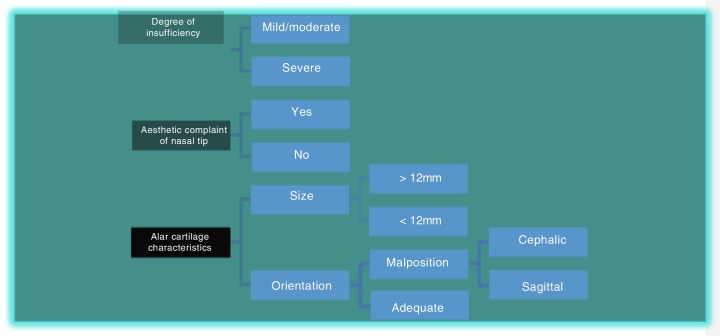
Initial parameters used to choose the appropriate treatment for the correction of external nasal valve insufficiency.

To choose the type of treatment for nasal valve insufficiency, we initially used three parameters as reference (Fig. 1):

Esthetic complaint of the nasal tip (present or absent);
Characteristics of alar cartilage (size and orientation);
Degree of external nasal valve insufficiency (mild, moderate, severe).

For surgeons performing rhinoplasty, the esthetic complaint of the nasal tip associated with external nasal valve insufficiency are important factors when choosing the treatment to be performed. Patients without esthetic complaint of the nasal tip allow us to perform the treatment without the exposure of nasal tip cartilage, which is performed through open rhinoplasty or closed rhinoplasty with delivery access. In these situations, one can place grafts through small incisions near the alar rim, with its extension being related to the size of the graft to be used. The alar rim grafts and the batten grafts are the available options.

Patients with esthetic complaint of the nasal tip associated with external nasal valve insufficiency should be submitted to procedures that expose the alar cartilages through open or delivery access (closed rhinoplasty). Overall, these complaints may be associated with the position of the nasal tip (underprojected or overprojected) or its shape (globose and/or asymmetric).

The size of the alar cartilages, specifically of the lateral crus, is also a condition that is evaluated for the choice of treatment option. We used the following reference parameter for the final configuration of the size of the lower lateral cartilages: 5 mm in the dome and 8 mm in the lateral crus region.^18^ Alar cartilages that have a lateral crus > 12 mm allow us to perform a maneuver that reinforces this structure without using grafts, by folding the cartilage on itself, called a “turn-in flap”. Cartilages < 12 mm do not allow us to perform this maneuver, since we must always maintain at least 8 mm in the lateral crus portion to prevent alar rim fragility.

The lateral crus orientation of the lower lateral cartilage is also essential in the diagnosis of external nasal valve insufficiency, since it will define the type of treatment chosen. Known as a cause of external valvular insufficiency and sometimes also as a cause of nasal tip esthetic complaint, the cephalically or “between parenthesis” lower lateral cartilage requires an adequate therapeutic approach. This anatomical alteration results in the absence of adequate support for the alar rim region. Among the treatment options are repositioning of the lateral crus with or without lateral crural strut graft,^19^ and some new options described in the literature as turn over flap.^20^ Another alteration of the lateral crus orientation of the lower lateral cartilage that can be found is the sagittal malposition, a condition identified with the depression of the caudal rim in relation to the cephalic rim. In this situation, the anomalous position of the caudal rim causes external nasal valve insufficiency.

The third parameter that was analyzed to define treatment is the degree of the external nasal valve insufficiency. No classification was found in the literature for this type of alteration. Therefore, we used mild-moderate insufficiency for patients with dynamic alterations of the external nasal valve (non-forced inspiration) or with a diagnosis of fragile and/or poorly positioned cartilages through inspection and palpation of the nose. Severe insufficiency is defined as those patients with collapse of the alar rim at static inspection of the external nasal valve, or patients with total or partial absence of lateral crus caused by iatrogenesis or malformation. These situations require more specific choices of graft type to be used. The following are options: batten graft, lateral crural strut graft, articulated alar rim graft or butterfly graft, all described in the literature.

**Figure 2:**
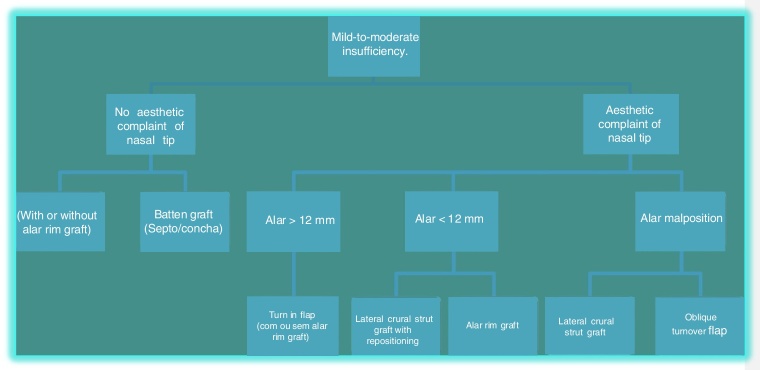
Algorithm for the treatment of mild-to-moderate external nasal valve insufficiency.

**Figure 3:**
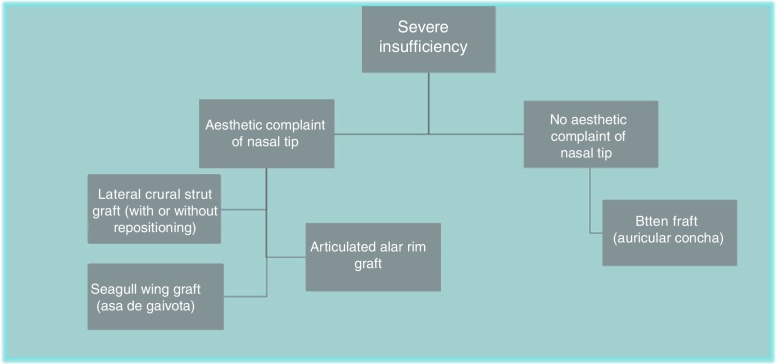
Algorithm for the treatment of severe external nasal valve insufficiency.

Therefore this treatment algorithm was created for external nasal valve insufficiency treatment (Figure 2, Figure 3).

**Figure 4:**
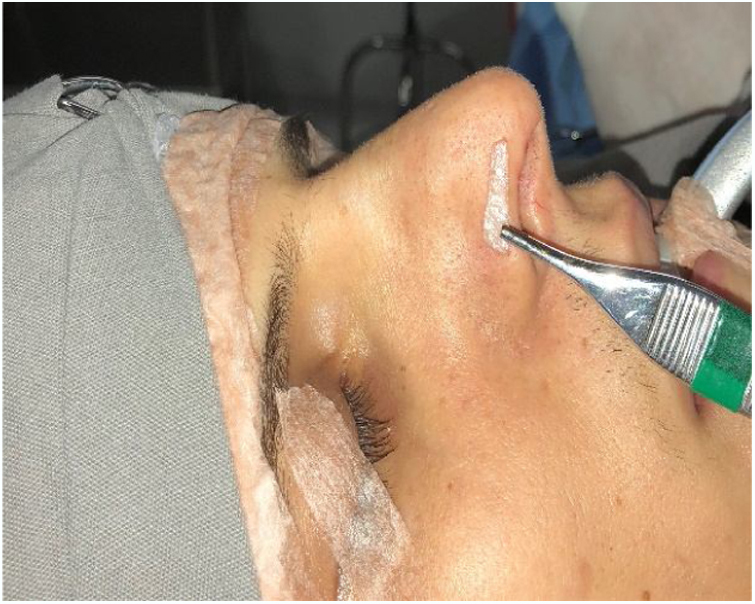
Alar rim graft created from septal cartilage. Place where the graft will be inserted.

**Figure 5:**
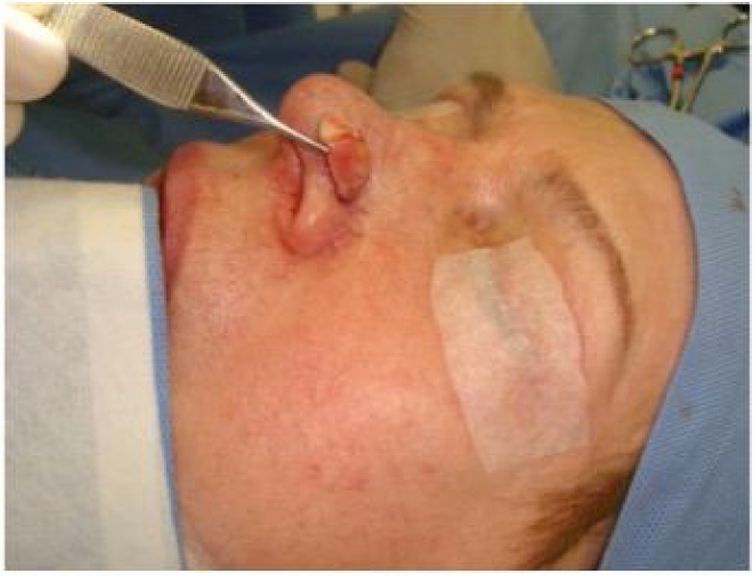
Batten graft created with conchal cartilage. Place where the graft will be inserted.

**Figure 6:**
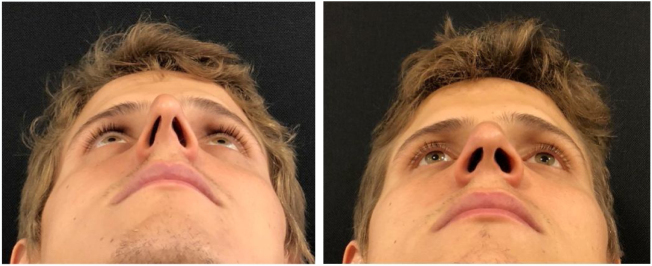
Pre and postoperative (6 months) periods of functional closed rhinoseptoplasty with bilateral alar rim graft, without access to the nasal tip.

The treatment options for patients with mild-moderate external nasal valve failure without esthetic complaint of the nasal tip are the alar rim graft^11^ (contour grafting) or batten graft.^10^ In these situations, the septal cartilage is the first choice; however, conchal cartilage can also be used. The alar rim graft should be positioned close to the alar rim (Fig. 4), whereas the batten graft should be located on the lateral crus (or its remnant) and extend to the piriform opening. For these options, we make a small incision near the caudal margin of the lower lateral cartilage and dissect a narrow space to receive the graft (Fig. 5). It is not necessary to fix it with sutures, as we do not perform a wide dissection of the region. Then, the incision is sutured with 1–2 simple absorbable stitches (Case 1) (Fig. 6).

**Figure 7:**
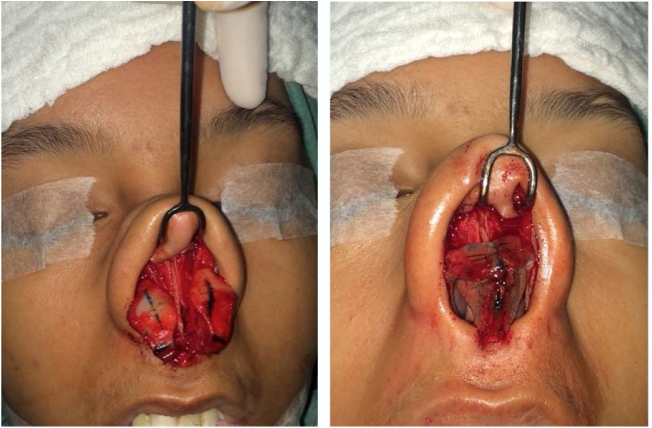
Intraoperative period. Turn in flap maneuver. Initially marked 8 mm on the lateral crus from the caudal border and then the cephalic portion is folded under the caudal remnant.

In patients with mild-to-moderate insufficiency and esthetic complaint of the nasal tip, the treatment options will be based on the characteristics of the alar cartilages. Adequately oriented alveolar cartilages that have a lateral crus size greater than 12 mm, can be treated with turn-in flap^14^ a maneuver that obviates the need for a graft, and consists of overlapping at least 4 mm of the lateral crus, thus generating greater stability in this region (Fig. 7).

**Figure 8:**
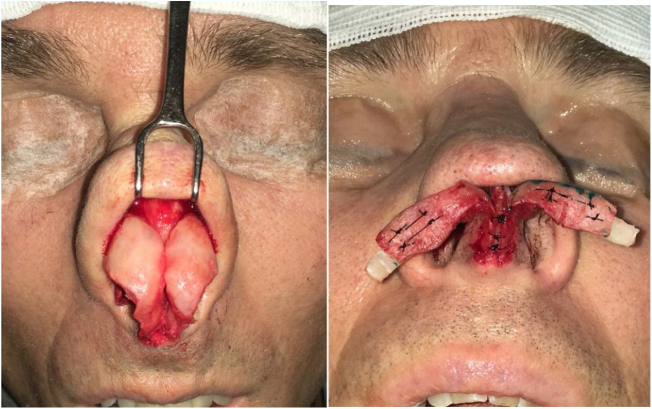
Intraoperative period. Patient with poorly positioned alveolar cartilages in the cephalic orientation. Complete detachment of the lateral crura and lateral crural strut graft fixation under the alar cartilages was performed.

**Figure 9:**
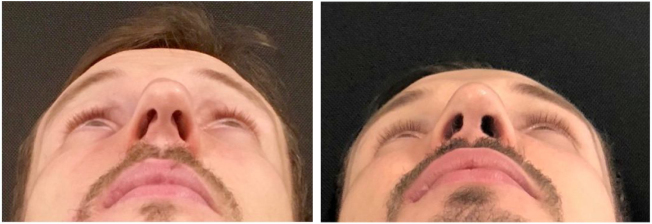
Pre- and postoperative (1 year) periods of functional rhinoseptoplasty and open esthetics, using lateral crural strut graft and lateral crura repositioning.

**Figure 10:**
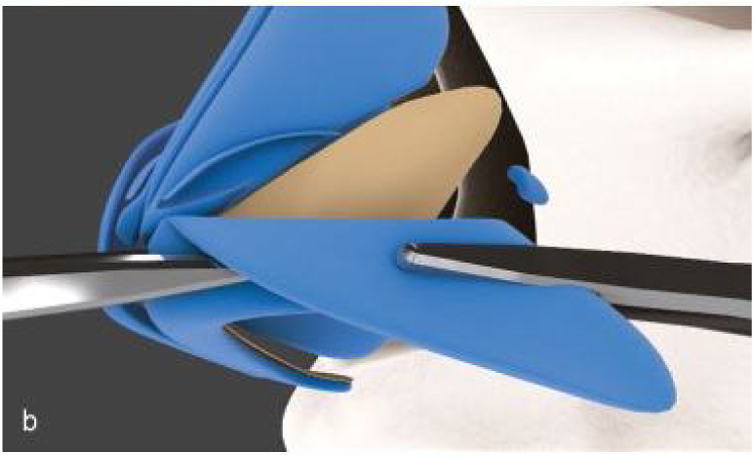
Image of oblique turnover flap for repositioning and flattening of the lateral crura. Goksel, Vladykina.

In situations with inadequately orientated lower lateral cartilages, regardless of their size, we must use other treatment options for this correction. Cartilages with poor cephalic or sagittal positioning can be treated with lateral crus repositioning, associated with the use of the lateral crural strut graft^13^ (Fig. 8). In this treatment, we detach the entire lateral crus, since the domus, from the underlying mucosa, and remove the lateral crus from the sesamoid cartilages. Next, a graft is fixed (lateral crural strut graft) on this lateral crus and these structures are repositioned to a new, lower position, with the detachment of a narrow tunnel next to the piriform aperture (Case 2) (Fig. 9). Another option for the correction of cephalic cartilage is the oblique turnover flap,^20^ described more recently in the literature. This maneuver consists of folding the lateral crus on itself at an oblique axis, changing its position and reinforcing the region of the alar rim (Fig. 10).

In patients with severe external nasal valve insufficiency, significant nasal obstruction was found due to this alteration. These situations are very often associated with previous surgeries, with aggressive resection of the lateral crus of the lower lateral cartilage, or malformation of these cartilages. In these cases, the options require a significant strengthening of the external nasal valve area, which will also have as criterion the observed aspect of the lower lateral cartilages. Some patients have only a functional complaint, and in these cases, the use of batten graft with auricular conchal cartilage is an excellent option, without the need for a wide access, but only the creation of a narrow area to place the graft.

**Figure 11:**
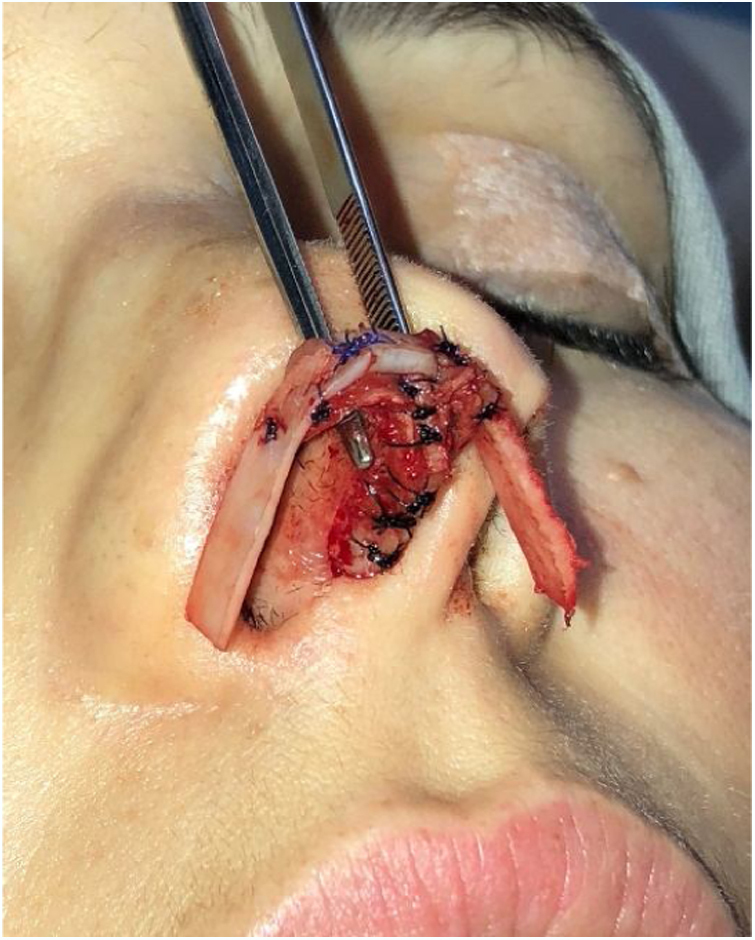
Intraoperative period. Articulated alar rim graft was used in primary closed rhinoseptoplasty, with graft fixation on the lateral crus near the domus.

**Figure 12:**
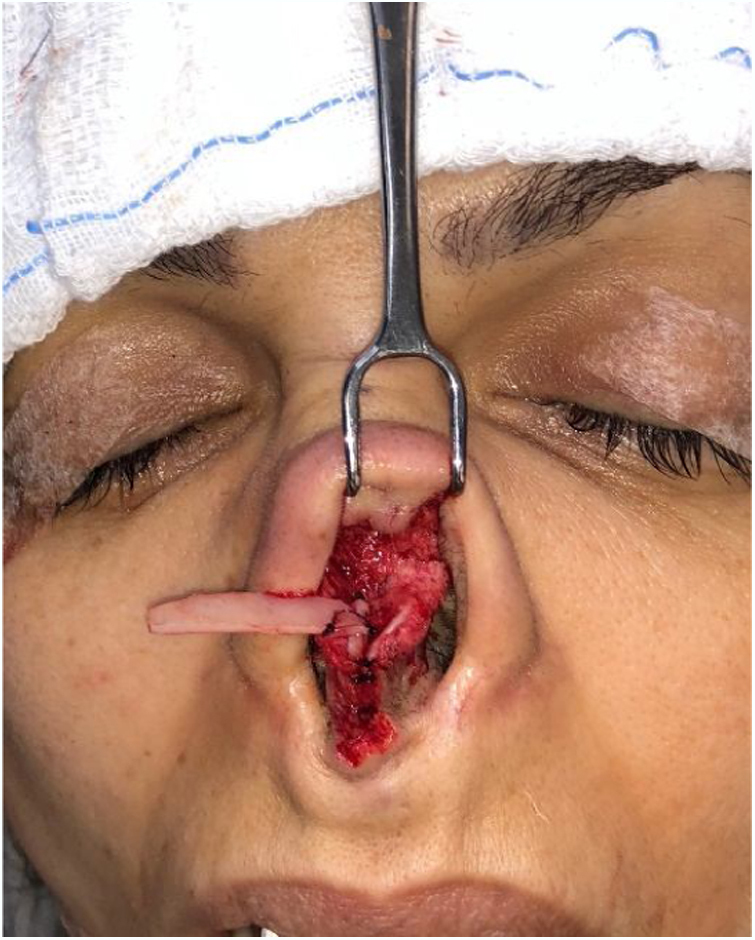
Intraoperative period. Articulated alar kidney graft used in open revision rhinoseptoplasty for external nasal valve remodeling. Patient had undergone 2 previous surgical procedures. The graft was attached to the right and, on the left, we can identify the remnant of the lower lateral cartilage with the previously amputated lateral crus. Subsequently, we also fixed the graft to the left.

**Figure 13:**
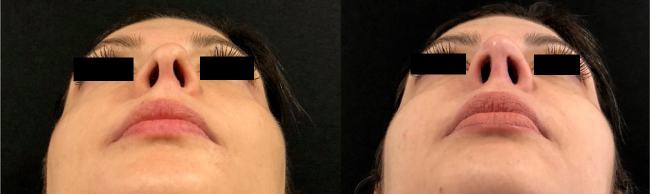
Pre- and postoperative (3 months) periods of esthetic and functional revision open rhinoseptoplasty, with the use of an articulated alar rim graft. Patient had undergone 2 previous nasal surgeries.

**Figure 14:**
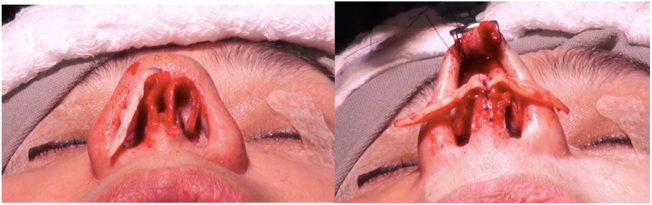
Intraoperative period. Seagull wing graft before being fixed (only shown the right graft). Subsequently, the grafts were positioned bilaterally on the remnants of the alar cartilages.

However, many patients also have esthetic alterations due to deformities in the lateral crus. In these situations, we can also use the previously described lateral crural strut graft. Another increasingly used option, is the articulated alar rim graft^12^ (Figure 11, Figure 12) that can be employed for these more severe situations. It is created preferably with septal cartilage (or costal cartilage), having its medial portion attached to the lower border of the lateral crus remnant near the domus and its most lateral portion is embedded into a new dissected pouch close to the piriform aperture, to stabilize this graft and give support to the alar rim (Case 3) (Fig. 13). In cases where we find partial or total resection of the lateral crus, we can use the seagull wing graft^15^ for the reconstruction of this portion of the lower lateral cartilage. In these cases it is necessary to use auricular conchal cartilage, as it has intrinsic concavities that are similar to the lateral crus (Fig. 14).

## Conclusion

The use of this simple and practical algorithm allows the use of each type of graft and/or maneuver according to the patients’ complaints and the anatomical alterations found in the inferior lateral cartilages for the correction of external nasal valve insufficiency.

## Conflicts of interest

The authors declare no conflicts of interest.
